# Autoimmune liver disease and the enteric microbiome

**DOI:** 10.3934/microbiol.2018.2.334

**Published:** 2018-05-14

**Authors:** Kerri Glassner, Eamonn MM Quigley, Lissa Franco, David W Victor

**Affiliations:** 1 Lynda K and David M Underwood Center for Digestive Disorders, Division of Gastroenterology and Hepatology, Houston Methodist Hospital, Weill Cornell Medical College, 6550 Fannin Street, SM 1201, Houston, TX 77030, USA; 2 Sherrie and Alan Conover Center for Liver Disease, Houston Methodist Hospital, Weill Cornell Medical College, 6550 Fannin Street, SM 1201, Houston, TX 77030, USA

**Keywords:** primary sclerosing cholangitis, primary biliary cholangitis, autoimmune hepatitis, microbiome, PSC, PBC

## Abstract

The human enteric microbiome is highly complex and has more than 150 times more genes within it than its host. The host and the microbiome have a commensurate relationship that can evolve over time. The typically symbiotic relationship between the two can become pathogenic. The microbiome composition in adults reflects their history of exposure to bacteria and environmental factors during early life, their genetic background, age, interactions with the immune system, geographical location, and, most especially, their diet. Similarly, these factors are thought to contribute to the development of autoimmune disease. It is possible that alterations in the intestinal microbiome could lead to liver disease. There is emerging data for the contribution of the microbiome in development of primary sclerosing cholangitis, primary biliary cholangitis, and autoimmune hepatitis; liver disorders associated with aberrant immune function in genetically susceptible individuals.

## 1. Introduction

The human enteric microbiome is made up of approximately 2–5 × 10^12^ bacteria per gram of fecal matter [1]. The microbiome is highly complex and has more than 150 times more genes within it than its host [2]. The relationship of the host and microbiota is not static. The symbiotic relationship can become pathogenic. The microbiome composition in adults reflects their history of exposure to bacteria and environmental factors during early life, their genetic background, age, interactions with the immune system, geographical location, and, most especially, their diet [3]–[8]. Similarly, these factors are thought to contribute to the development of autoimmune disease. The liver receives a significant amount of its blood supply from the splanchnic circulation, and thus is exposed to bacteria and bacterial products from the intestinal microbiome [9]. It is possible that alterations in the intestinal microbiome could lead to liver disease. This review will focus specifically on the role of the microbiome in the development of primary sclerosing cholangitis, primary biliary cholangitis, and autoimmune hepatitis; liver disorders associated with aberrant immune function in genetically susceptible individuals.

## 2. Primary sclerosing cholangitis

Primary sclerosing cholangitis (PSC) is a progressive immune-mediated liver disease that leads to fibrosis of the intra- and extra-hepatic bile ducts with chronic cholestasis and often end-stage liver disease. PSC patients are at higher risk for the development of cholangiocarcinoma, hepatocellular carcinoma and colon cancer. Previous studies have indicated that anywhere from 60 to 80% of patients with PSC also have concomitant inflammatory bowel disease (IBD) [10]. Recent studies have supported the role of the intestinal microbiome in the pathogenesis of PSC. While the exact mechanism is unclear, these studies have explored the role of antibiotics, fecal microbiota transplantation (FMT), and diet in altering the microbiome in PSC patients.

### 2.1. PSC and association with IBD

The strong association of PSC with IBD is suggestive of a link between the gut microbiome and the liver. In the “microbiota hypothesis”, microbial molecules arising from the intestine reach the liver through the portal circulation, and initiate a cholangiocytic response [11]. This is thought to be due to intestinal inflammation allowing translocation of enteric pathogens through the mucosal barrier. Another possibility is the “gut lymphocyte homing” hypothesis. Activated T cells from the intestine are thought to hone in on the liver and initiate immune-mediated damage [10].

Evidence has emerged of a distinct gut microbial profile in patients with PSC, separate from those with ulcerative colitis (UC). In a study of 85 patients with PSC, 16S rRNA gene sequencing showed reduction of bacterial diversity in PSC patients compared with healthy controls. This reduction was independent of the presence of UC [12]. *Veillonella* sp. was found in greater abundance in patients with PSC compared to healthy controls or to those with IBD without PSC. Another study of 43 PSC patients similarly found PSC to be associated with specific gut microbial signatures independent of concomitant IBD. PSC with or without IBD was associated with increased *Rothia*, *Enterococcus*, *Streptococcus*, *Clostridium*, *Veillonella*, and *Haemophilus* species, compared to UC alone. Both PSC and UC patients had decreased bacterial diversity and changes in global microbial composition [13]. A third study by Sabino et al [14] also showed that in 52 PSC patients there was significantly different fecal microbiota composition compared to healthy controls and patients with IBD without PSC. Microbial diversity was lower in PSC patients with ulcerative colitis compared to healthy controls. Three genera were identified to be associated with PSC: *Enterococcus*, *Lactobacillus*, and *Fusobacterium* species, and this association was independent of such potential confounders as IBD, probiotic use, liver cirrhosis, liver transplantation, ursodeoxycholic acid use or antibiotic treatment. In fact, *Enterococcus* sp. abundance was found to correlate with the level of alkaline phosphatase in serum on univariate analysis.

The specific alteration of signatures seen in the prior studies suggests that changes in the microbiome distinctly influence the development of PSC. Using a mouse model the cytoprotective effect of the microbiota has been suggested. The germ free mice were found to have more abnormal serum biochemistries, absent secondary bile acids, increased severity of fibrosis, ductular reaction, ductopenia, and increased cholangiocyte senescence [15]. Bacterial metabolites may protect against injury-induced cholangiocyte senescence, explaining why the germ free mice have more severe disease. Cholangiocyte senescence has previously been found to be associated with PSC. Persistent exposure of cholangiocytes to various microbial molecules such as LPS and flagellin induced human cholangiocyte senescence. Increased makers of cholangiocyte senescence were also seen in PSC [16],[17]. In the same study, UDCA, a secondary bile acid produced by commensal bacteria, was found to decrease the amount of cholangiocyte senescence, suggesting the microbiota may be cytoprotective against biliary injury. The combination of these findings is very suggestive for the role of an altered microbiome in the development of PSC, which may include a protective role for a normal commensal microbiota, and a pathogenic role for some bacterial species.

### 2.2. PSC and antibiotics

Antibiotic use is known to lead to changes within the enteric microbiome, which can predispose to the development of infections such as *Clostridium difficile* colitis. On the other hand, antibiotics are also used in the treatment of enteric infections as well as other gastrointestinal tract conditions including small bowel bacterial overgrowth and irritable bowel syndrome. The use of antibiotics has been looked at as both a potential factor in the development of PSC and as a treatment strategy.

Eaton et al. studied one thousand patients with PSC and found that women with PSC were more likely to have experienced frequent urinary tract infections, regardless of the presence of IBD[18]. One explanation for this observation is that the use of antibiotics for recurrent urinary tract infections alters the microbiome, thereby, predisposing to the development of PSC. However, it is not clear whether these women had repeated urinary tract infections prior to the diagnosis of PSC or only after diagnosis. Conversely, it may be that the altered microbiome present in PSC leads to repeated urinary tract infections.

In the early1990's, Lichtman et al. classically demonstrated that rats who had undergone small bowel ligation to cause small bowel bacterial overgrowth subsequently developed biliary ductal dilatation and beading resembling PSC. Interestingly, daily treatment with antibiotics resulted in near-complete histological improvement [19]. More recently, the use of antibiotics in the treatment of human patients with PSC has been explored. Vancoymcin, metronidazole, tetracycline, sulfasalazine, azithromycin, and minocycline have all been found to improve laboratory parameters [20]–[32]. In one study, 35 patients with PSC were randomized to either high or low dose vancomycin or metronidazole for 12 weeks [21]. Patients treated with low dose vancomycin achieved the primary endpoint of a decrease in alkaline phosphatase (ALP) at 12 weeks, and were also more likely to achieve normalization of ALP, as well as a decrease in Mayo PSC risk score, total bilirubin and c-reactive protein (CRP). There was also an improvement in bilirubin and the Mayo PSC risk score with metronidazole. The microbiota hypothesis suggests that bacterially derived molecules such as LPS, lipoteichoic acid and DNA fragments enter the portal circulation through a breach in the enteric mucosal barrier and contribute to hepato-biliary inflammation. PSC has been shown to be associated with portal bacteremia, bacterobilia, and 16S ribosomal ribonucleic acid in bile [33]–[35]. In addition, cholangiocytes in PSC accumulate LPS [36]. The improvement seen with antibiotic treatment could be explained by changes in the microbiome resulting in decreased synthesis of inflammatory molecules, modifying the hepatobiliary immune responses and signaling cascades.

Several other studies have found similar results. Farkkila et al. [23] compared 80 patients with PSC randomized to either UDCA alone or in combination with metronidazole. Patients treated with both UDCA and metronidazole had improvement in ALP levels and Mayo risk score; however, there was no significant effect on disease progression based on liver histology and ERCP findings. Another study of 14 children with PSC and IBD who were treated with oral vancomycin found that there was normalization or improvement in liver enzymes, inflammatory markers, and clinical symptoms in nearly all of the patients [32]. When vancomycin was discontinued, symptoms worsened and liver enzymes in increased in several patients only to again improve on retreatment with vancomyin. The use of probiotics has also been studied. Fourteen patients with PSC and IBD who were treated with a probiotic cocktail containing *lactobacillus* and *bifidobacterium* strains did not show any improvement in clinical symptoms or laboratory parameters [37].

### 2.3. PSC and fecal transplant

Fecal Transplantation (FMT) dramatically alters the host microbiome. A recent study investigated the role of FMT in PSC. This study was small and enrolled just 10 patients of whom only 6 underwent fecal transplant. Three of the six who underwent FMT had a 50 percent or greater decrease in alkaline phosphatase by week 26. The average decrease in the responders was 250 units compared to 10 among non-responders. The microbiome was analyzed prior to FMT and revealed significantly reduced diversity compared to controls with increased numbers of *Enterobacteriales* and *Fusobacterium* species. Diversity was increased in all PSC patients at week one post FMT and remained elevated in the majority [38]. In post FMT samples, *Fusobacterium* sp. remained elevated and *Enterobacteriales* sp. decreased non-significantly. This data is intriguing; however, larger studies are needed to further elucidate the role of FMT in PSC patients.

### 2.4. PSC and the role of diet

It is well established that dietary changes can affect the microbiome [39]. Several studies have investigated the association between diet and PSC. One study found that patients with PSC were less likely to be daily coffee drinkers than controls [40]. Of 480 patients with PSC evaluated using a questionnaire, 24% reported that they had never consumed coffee compared with 16% of controls, and only 67% were current drinkers compared with 77% of controls. In this study, coffee was also protective against proctocolectomy in PSC patients with ulcerative colitis [41]. PSC patients have been found to be less likely to consume fish, but more likely to consume steak or well-cooked hamburgers [17]. Another study investigated the specific changes that dietary factors have on the microbiome in PSC [42]. *SMB53* genus was correlated with alcohol intake and *Bifidobacterium* sp. with omega-6 fatty, *Enterobactericae* sp. with boron, and *Parabacteroides* sp. with manganese consumption. These findings suggest that dietary and cooking habits may contribute to the development of PSC through alterations in the gut microbiome. Alternatively, dietary changes associated with PSC could lead to the observed microbiota changes.

## 3. Primary biliary cholangitis

Primary Biliary Cholangitis (PBC) is the most common autoimmune liver disease, with an incidence of 0.33 to 5.8 per 100,000 and prevalence of 2 to 40.2 per 100,000. The prevalence appears to be increasing [43]. This is thought to be due to increasing incidence, advances in diagnosis, and slow progression of disease [44]. Another factor is the introduction of successful treatment with ursodeoxycholic acid, prolonging survival without cure, thus increasing prevalence [45]. The disease is characterized by elevated alkaline phosphatase, a positive anti-mitchondrial antibody (AMA) in 95% of patients, and chronic non-suppurative cholangiopathy on liver biopsy. There is chronic damage to the small intralobular bile ducts by a T-lymphocyte mediated attack, which leads to chronic progressive cholestasis [46]. Disease progression can eventually lead to cirrhosis and liver failure. There is evidence that the microbiome may play a role in the pathogenesis of PBC.

### 3.1. Gut liver axis in PBC

PBC can alter the enteric microbiome by altering intestinal motility, immunologic derangement, bile secretory defects and portal hypertension [44], [47]–[49]. A study of 42 patients with PBC demonstrated that individuals with PBC have fewer *Acidobacteria*, *Lachnobacterium*, *Bacteroides*, and *Ruminococcus* species. There were higher numbers of microbes considered to be opportunistic pathogens including *Y-Proteobacteria*, *Enterobacteriaceae*, *Neisseriaceae*, *Spirochaetaceae*, *Veillonella*, *Streptococcus*, *Klebsiella*, and *Actinol* species. PBC patients were found to have higher levels of certain cytokines including IL-8, IL-18, IL-16, IP-10, MIG, IL-2RA, and Macrophage migration inhibitory factor (MIF). In fact, microbes less abundant in PBC patients in comparison to healthy controls were negatively associated with levels of inflammatory cytokines. For example, *Ruminococcus* sp. were more abundant in healthy controls than in PBC, and associated with lower levels of IL-16 and monokine induced by interferon gamma (MIG) [50]. These findings imply that alterations within the microbiome in PBC patients may have an impact on immune function. Along these lines, PBC has also been associated with the abnormal accumulation of lipopolysaccharide (LPS) in biliary epithelial cells [51]. LPS is in the outer membrane of gram-negative bacteria, and prior studies have shown that exposure to LPS elicits a strong host immune response [52]. Polymorphisms in the gene coding for toll-like receptor (TLR) 4 (whose natural ligand is LPS) have been identified in PBC. The polymorphisms in TLR-4 may lead to accumulation of LPS in biliary epithelial cells, which may suggest a strong host immune response to the accumulation. This could further support a link between microbial products, the immune system, and PBC [53]–[55].

### 3.2. PBC and ursodeoxycholic acid

Bile acids are important metabolites of the microbiome and can modulate its composition directly or through the activation of the innate immune system [56]. Conversely, the gut microbiome is the key facilitator of the enterohepatic circulation of bile acids [57]. The 95% of bile acids secreted by the liver and excreted into the intestine are reabsorbed by the intestinal epithelium and transported back through the portal vein to the liver, where they are conjugated and secreted into bile; the 5% of the total bile acid pool, which is not absorbed, undergoes deconjugation by the colonic microbiota [45]. Alterations in the microbiome may result in changes in bile acid composition and circulation contributing to PBC.

Ursodeoxycholic acid (UDCA) can improve liver biochemistry, delay disease progression and even prolong survival in PBC [58], [59]. *Ruminococcus* sp., found to be decreased in patients with PBC, is able to form UDCA [60]. It is quite possible that changes in the microbiome such as decreased numbers of *Ruminococcus* sp., may lead to alterations in the production and enterohepatic circulation of bile acids, potentially contributing to the development of PBC. A recent study performed in treatment-naïve patients with PBC, also found substantial differences in the composition and function of the gut microbiome compared with healthy controls. *Bacteroidetes* sp. was the most abundant genus in both groups, and was decreased in PBC compared to a healthy control group. *Sutterella*, *Oscillospira* and *Faecalibacterium* species were enriched in healthy controls. In the PSC group, *Haemophilus*, *Veillonella*, *Clostridium*, *Lactobacillus*, *Streptococcus*, *Pseudomonas*, *Klebsiella* species and an unknown genus in family of *Enterobacteriaceae* were increased. The use of UDCA was found to partially reverse the differences in the genera identified between healthy controls and treatment naïve PBC patients after six months of treatment [61]. *Haemophilus*, *Streptococcus*, and *Pseudomonas* species decreased after UDCA treatment in PBC patients. *Bacteroidetes*, *Sutterella* and *Oscillospira* species, which were enriched in the healthy control group compared to the PBC group, increased in the PBC group post treatment with UDCA. Interestingly, *Veillonella* sp., which was more abundant in PBC patients, was significantly more prevalent in treated patients who had an inadequate response to UDCA. These findings are suggestive of an important interaction between bile acids and the microbiome in patients with PBC.

### 3.3. PBC and anti-mitochondrial antibodies

There have been several studies investigating the role of infectious agents as initiators of an autoimmune assault on the biliary epithelium [45]. These have shown, for example, that anti-mitochondrial antibodies exhibit cross-reactivity to antigens from the bacteria *Escherichia coli* and *Novosphingobium aromaticivorans* [62]–[64]. Infection or colonization with *E. coli* or *N. aromaticivorans* likely incites an immune response that, in genetically susceptible individuals, can trigger the development of anti-mitochondrial antibodies through molecular mimicry. Mice that are inoculated with *N. Aromaticivorans* develop PBC-like disease, further supporting this theory [65].

A study by Smyk et al [66] found that urinary tract infections are a risk factor for the development of PBC. *Klebsiella* sp., a common cause of urinary tract infection, has been implicated in the pathogenesis of several autoimmune disorders, likely due to molecular mimicry and cross-reactivity [67]. An unknown genus in the family of Enterobacteriaceae was shown to have an association with PBC and the presence of AMA [68]. It is possible that Klebsiella sp., also a genus in the family Enterobacteriaceae, may lead to molecular mimicry, cross-reactivity and induction of anti-mitochondrial antibodies in susceptible individuals with recurrent *Klebsiella* sp. urinary tract infections. *Klebsiella* sp. has also been found to have a positive correlation with serum levels of bilirubin in patients with PBC [62]. It appears that infection or colonization with specific bacteria, possibly from an altered intestinal microbiome, can trigger development of anti-mitochondrial antibodies through molecular mimicry and lead to PBC.

Several lines of evidence support a role for the microbiota in IBS but the relative contributions of a disturbed microbiota, per se, changes in bile salt metabolism and molecular mimicry between bacteria and the biliary epithelium have not been defined.

## 4. Autoimmune hepatitis

Autoimmune hepatitis (AIH) is a chronic, progressive, immune-mediated inflammatory liver disease. The disease predominantly affects females, and is characterized by elevated immunoglobulin levels and transaminases, the presence of autoantibodies and the development of interface hepatitis [69]. The annual incidence is 0.69–1.9 cases per 100,000 people with a prevalence of 4–42.9 cases per 100,000 people [70]–[75]. Its pathogenesis has been associated with several factors including, genetic pre-disposition, breakdown of immune tolerance, and exposure to environmental factors which trigger the autoimmune process and result in a T cell mediated response to liver antigens [76]. This, in turn, leads to inflammatory activity and tissue destruction. Studies have shown an association of the intestinal microbiome with the development of AIH, as with PSC and PBC. Overlap syndromes with PSC or PBC occur in 7–18% of patients [77],[78], supporting this link between diseases. Recent studies have suggested that AIH is associated with an altered microbiome, increased permeability of the intestinal mucosal barrier, and translocation of both microbes and microbial products from the gut into systemic circulation [76].

### 4.1. AIH and intestinal permeability

In AIH, tight junctions in the intestinal mucosa may weaken and allow translocation of lymphocytes, bacterial ligands and endotoxin across the mucosal barrier [76]. One study found that compared to healthy controls, patients with AIH have duodenal villi that are small, scarce, and irregular in arrangement with disrupted tight junctions. In fact, zona occludens 1 and occludin, structural proteins which function in the binding of intestinal epithelial cells, are reduced in patients with AIH compared to healthy volunteers [79], [80]. With more advanced AIH liver disease, there was decreased expression of occludin and zona occludens 1. The same study also found that within the enteric microbiome, there are reduced anaerobes (*bifidobacterium* and *lactobacillus* species) in patients with AIH.

Previous studies have also shown changes in the intestinal microbiome in experimental models of autoimmune hepatitis [81]. Yuksel et al developed a novel mouse model of AIH using the HLA-DR3 transgenic mouse on the non-obese diabetic (NOD) background by immunization with a DNA plasmid coding for human CYP2D6/FTCD fusion protein. HLA-DR3 is strongly linked to AIH. The HLA-DR3 positive mice that were immunized developed chronic liver injury that mirrored AIH. These mice were found to have a significantly different microbiome composition than the HLA-DR3 negative mice. The AIH mice had decreased diversity of microbiota with significantly less *firmicutes* sp. It is possible that changes in the microbiome, in combination with an altered mucosal barrier, may allow the translocation of bacterial products such as short chain fatty acids, endotoxin, LPS, and bacterial components that can serve as antigenic ligands, leading to an inflammatory response.

Supporting this theory, plasma LPS levels are increased in AIH, suggestive of bacterial translocation [79]. With more advanced AIH liver disease, the level of plasma LPS increases. Plasma LPS generated from translocation across the enteric barrier, can then enter the portal vein and liver with activation of toll-like receptors in hepatocytes, stellate cells, Kupffer cells, and sinusoidal epithelial cells. This results in the generation of pro-inflammatory cytokines and reactive oxygen species that lead to inflammation, predisposition to autoimmunity and fibrosis.

### 4.2. AIH and anti-neutrophil cytoplasmic antibody

Anti-neutrophil cytoplasmic antibodies (ANCA), are a group of autoantibodies, mainly in the IgG class, directed against antigens in the cytoplasm of neutrophil granulocytes and monocytes. Environmental factors and genetic predisposition are thought to contribute to their development [82]. Atypical p-ANCA is associated with PSC and autoimmune hepatitis. Studies have shown that the antigen B-tubulin that is targeted by atypical p-ANCA, cross-reacts with a bacterial antigen, FtsZ [83]. FtsZ is an evolutionary precursor protein of B-tubulin present in almost all bacteria of the intestinal microbiota. This suggests that molecular mimicry to bacterial products may also play a role in the development of AIH.

## 5. Summary

The relationship between the enteric microbiome and autoimmune liver disease continues to be an area of emerging research and potential clinical significance. It is increasingly evident that a microbiome-gut-liver axis exists, and could play a significant role in the development of autoimmunity in primary sclerosing cholangitis, primary biliary cholangitis, and autoimmune hepatitis. Evidence supports a multifactorial process, with genetics, and environmental triggers in the setting of an altered microbiome as contributing factors. While various alterations in the microbiota have been demonstrated in these autoimmune liver diseases, the precise role of the microbiome in any of these disorders is far from established. Not only must the contributions of various confounding factors be accounted for, but changes in the microbiome and gut barrier function described in these diseases could be secondary to rather than causative of, liver pathology. Exciting investigations are ongoing on the use of fecal transplantation, antibiotics, diet, probiotics and specific medications, such as ursodeoxycholic acid in PBC, to alter the microbiome and thus influence the course of disease activity.
